# Supplementary material for: Dynalign II: common secondary structure prediction for RNA homologs with domain insertions
Source: Nucleic Acids Res. 2014 Nov 21;42(22):13939–48. doi: 10.1093/nar/gku1172 (PMC4267632; doi:10.1093/nar/gku1172)
Supplement: SUPPLEMENTARY DATA [file supp_gku1172_nar-02021-z-2014-File012.zip › manual/javadoc/RNAstructure_java_interface/source/menus/package-frame.html]

RNAstructure\_java\_interface.source.menus


# RNAstructure\_java\_interface.source.menus

## Classes

- ConstraintsMenu
- DynamicMenuBar
- FileMenu
- HelpMenu
- NucleicAcidMenu
- RolloverMenu
